# The Curious Case of the Disappearing Cancer

**DOI:** 10.7759/cureus.73055

**Published:** 2024-11-05

**Authors:** Mahnoor Saad, Laila Babar, Sheza Khawaja, Muhammad Arqam Iqbal Goraya

**Affiliations:** 1 Internal Medicine, Howard University Hospital, Washington, DC, USA; 2 Oncology, University of Iowa Hospitals and Clinics, Iowa City, USA; 3 Internal Medicine, Weiss Memorial Hospital, Chicago, USA

**Keywords:** cancer, gerd, malignancy, screening, smoking

## Abstract

Esophageal cancer, particularly squamous cell carcinoma, poses significant diagnostic challenges due to its aggressive nature and similarity to metaplastic tissue. Accurate diagnosis often requires multiple biopsies and vigilant surveillance, especially in high-risk individuals with conditions such as gastroesophageal reflux disease (GERD) and a history of smoking.

We present a 66-year-old female patient with a history of severe GERD and smoking, who underwent routine endoscopy revealing a gastric cardia nodule. Although the initial biopsy showed benign results, a follow-up biopsy three months later indicated invasive well-differentiated squamous cell carcinoma. The patient underwent endoscopic resection, but the final pathology surprisingly revealed no malignancy. Ongoing surveillance has shown no recurrence.

This case underscores the complexities in differentiating between well-differentiated cancer and metaplastic tissue, as initial biopsies may not always reflect final pathology. Repeat biopsies are essential in suspicious cases to avoid misdiagnosis. For high-risk patients with GERD or Barrett's esophagus, early detection and regular monitoring are critical to prevent progression to esophageal cancer.

This case illustrates the importance of repeated diagnostic evaluations and careful surveillance to ensure accurate diagnosis and avoid overtreatment. It highlights the nuances of clinical medicine, where initial findings may not always tell the full story, emphasizing the need for a cautious, systematic approach to patient care.

## Introduction

It is important to recognize that metaplastic tissue and well-differentiated cancer can appear similar, necessitating multiple biopsies for a conclusive diagnosis. 

Implementing rigorous monitoring programs is crucial for individuals at high risk of esophageal cancer.

Since cancer represents a failure of the immune system, patients are at risk of developing multiple primary tumors, either at the same time (synchronous) or at different times (metachronous). Therefore, it is crucial to carefully evaluate these patients to ensure they do not have any additional underlying diseases [1].

## Case presentation

We present the case of a 66-year-old female patient with a significant medical history of severe gastroesophageal reflux disease (GERD), refractory to both pharmacologic treatment and lifestyle modifications. She also has a history of vocal cord paralysis for two years, along with hoarseness of voice. Notably, the patient had a prior history of smoking, which she discontinued in 1995, a factor that further elevated her risk for gastrointestinal malignancies. This patient also had a significant family history of lung cancer in both parents and two siblings.

The patient initially presented for worsening burning in her chest, attributed to GERD, and was put on a treatment of lifestyle modifications and proton pump inhibitors (PPIs). However, despite this, her GERD worsened. It is important to note that the patient also complained of worsening hoarseness. Of note, the patient had a previous history of right-sided vocal cord paralysis, for which she had already established care with otolaryngology. The patient was then referred to gastroenterology for further evaluation of GERD.

The patient underwent a routine colonoscopy and esophagogastroduodenoscopy (EGD) due to persistent GERD symptoms. While the colonoscopy was unremarkable, the EGD revealed esophagitis and a small nodule located in the gastric cardia. Although the initial biopsy of the nodule returned benign results, the visual appearance of the lesion raised sufficient concern for the gastroenterologist to recommend a repeat endoscopy after three months for further evaluation. 

During the follow-up EGD, a biopsy of the same area in the gastric cardia revealed pathology consistent with "invasive well-differentiated squamous cell carcinoma." The patient subsequently underwent endoscopic resection of the nodule. However, the final pathology from the resected tissue surprisingly showed no evidence of malignancy. Following this, the patient has remained under surveillance. 

**Figure 1:**
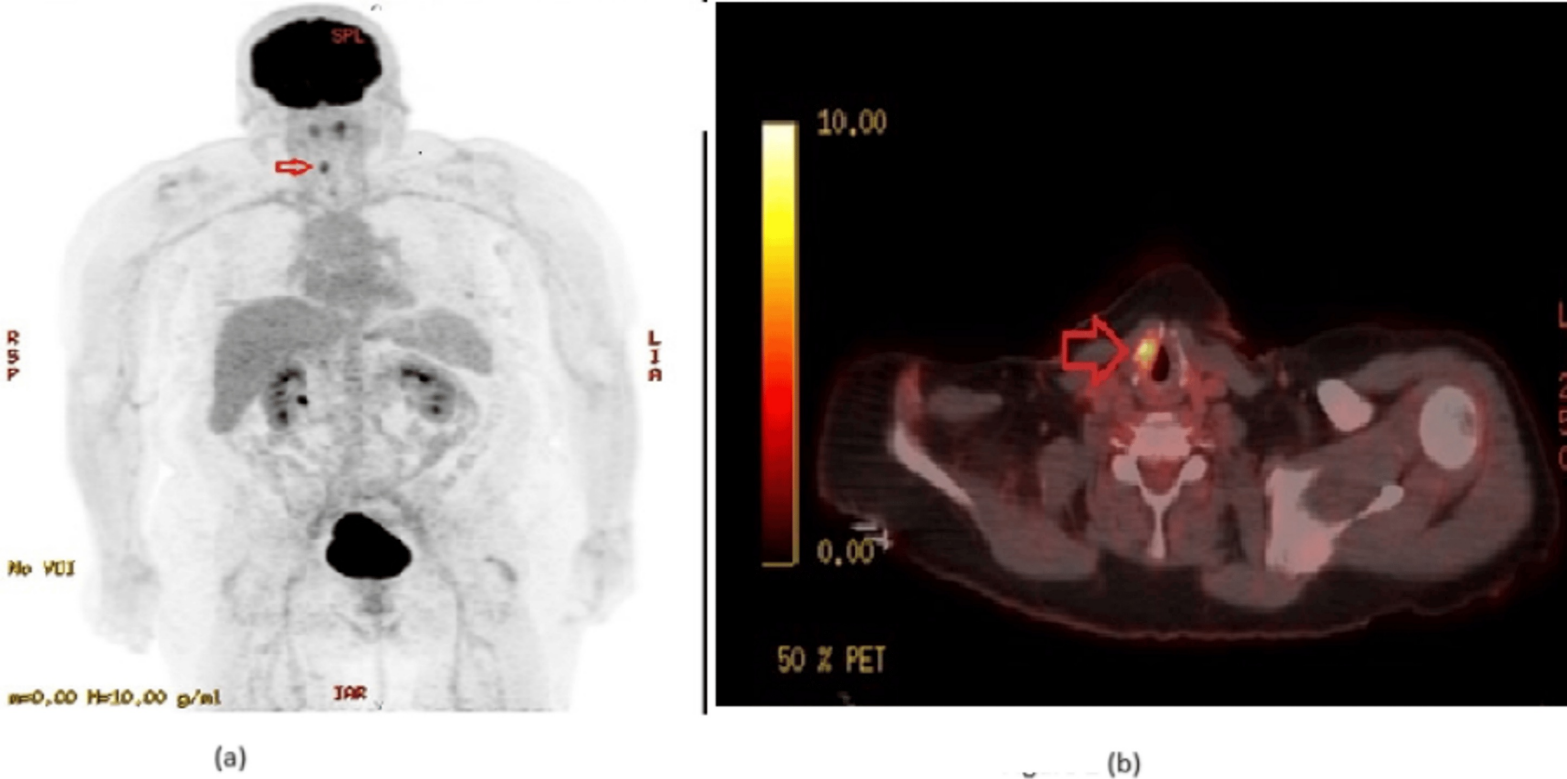
Fluorodeoxyglucose (FDG) avid right-sided vocal cord PET scan showing the reactive right-sided laryngeal node in (a) coronal plane and (b) transverse plane

**Figure 2:**
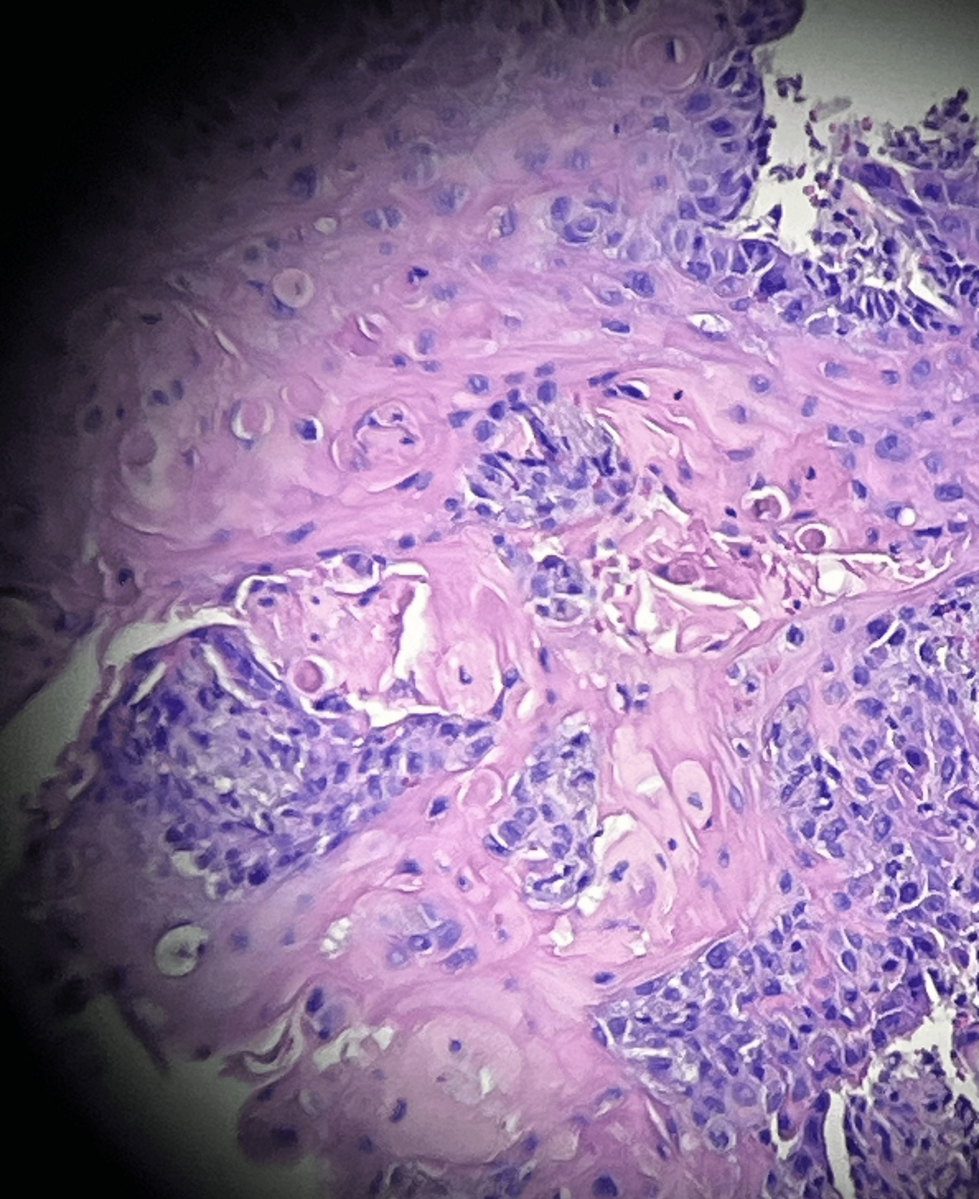
Invasive squamous cell carcinoma Magnification x50 Stain: Hematoxylin

Upon review of her positron emission tomography (PET) scan, she had fluorodeoxyglucose (FDG) avid right-sided vocal cord (Figure 1). Generally, an FDG-positive vocal cord is considered false positive, as it could be due to a paralyzed vocal cord like in this patient, or an artifact due to speaking during the FDG uptake phase, or could be a benign lesion in a vocal cord, which is also very common. It rarely represents true malignancy. Per one retrospective review, only 2.2% of FDG avid asymmetric vocal cord uptake was found to be malignant [2]. However, due to her history of positive esophageal biopsy, after discussion with her otolaryngologist, the vocal cord site was biopsied, and she was found to have squamous cell carcinoma of the larynx (Figure 2).

The patient was staged as T3 N0 M0, as the vocal cord was fixed on presentation. 

The treatment plan is chemotherapy and radiation. The patient is currently undergoing chemotherapy with weekly cisplatin of 40mg/m^2^

## Discussion

Esophageal cancer remains a significant global health concern due to its aggressive nature and poor prognosis when diagnosed at advanced stages. In this case, we highlight the diagnostic challenges posed by the similarity between well-differentiated cancer and metaplastic tissue. A single suspicious biopsy does not necessarily confirm malignancy, as subsequent resections may reveal benign pathology. This emphasizes the importance of repeat biopsies in uncertain cases to avoid premature or incorrect diagnoses. Moreover, vigilant surveillance is crucial, particularly for high-risk patients with conditions such as GERD or Barrett’s esophagus. Both GERD and Barrett’s are known precursors to esophageal adenocarcinoma, making it essential to closely monitor these patients through endoscopic evaluations and timely interventions. Early identification and treatment of pre-malignant conditions can significantly reduce the risk of cancer development. Therefore, comprehensive management of GERD and Barrett’s esophagus, whether through medical therapy or endoscopic interventions, plays a key role in preventing progression to cancer. This case underscores the need for a cautious, thorough approach to diagnosis and surveillance, ensuring that early-stage cancer is not missed, and benign lesions are not over-treated. This case also highlights the need for surveillance of awareness about the possibility of other malignancies being present in the patient [3-6].

Although the mainstay of treatment for both laryngeal and esophageal cancers is chemotherapy and radiation [7], the chemotherapy backbone in both cases is very different, with cisplatin-based chemotherapy for laryngeal [8] and carboplatin with paclitaxel for esophageal carcinoma [9].

It is important to note that even though laryngeal cancer risk is decreased by 70% 10 years after smoking cessation, it is never zero. Our patient demonstrated an occurrence despite quitting 30 years ago [10].

However, due to new technology and the integration of AI in diagnosing esophageal squamous cell carcinoma, we may see significantly enhanced detection rates, particularly in high-speed endoscopic videos, where traditional methods may fall short. By achieving high sensitivity rates, AI not only aids in early diagnosis but also supports endoscopists in improving their diagnostic accuracy, potentially leading to better patient outcomes​ [11].

## Conclusions

This case highlights the inherent complexities and uncertainties in clinical medicine, emphasizing the importance of thorough, repeated evaluations in the diagnostic process, as well as keeping an eye out for different malignancies. Despite advancements in diagnostic techniques, medicine remains filled with gray areas where visual impressions and initial biopsy results may not always align with final pathology; however, advances in AI may help with that. As clinicians, it is imperative to maintain a high index of suspicion and recognize that one set of findings may not tell the full story. Repeat biopsies, further diagnostic interventions, and vigilant surveillance are often necessary to ensure accurate diagnoses and appropriate patient management. Ultimately, this case underscores the value of meticulous attention to detail and the importance of verifying initial findings before committing to definitive diagnoses or treatment plans. In the ever-evolving landscape of medicine, a cautious and systematic approach remains paramount in delivering the highest standard of patient care. 
